# MESSI: metabolic engineering target selection and best strain identification tool

**DOI:** 10.1093/database/bav076

**Published:** 2015-08-08

**Authors:** Kang Kang, Jun Li, Boon Leong Lim, Gianni Panagiotou

**Affiliations:** ^1^Systems Biology & Bioinformatics Group, School of Biological Sciences, The University of Hong Kong, Pokfulam Road, Hong Kong and; ^2^School of Biological Sciences, The University of Hong Kong, Pokfulam Road, Hong Kong

## Abstract

Metabolic engineering and synthetic biology are synergistically related fields for manipulating target pathways and designing microorganisms that can act as chemical factories. *Saccharomyces cerevisiae*’s ideal bioprocessing traits make yeast a very attractive chemical factory for production of fuels, pharmaceuticals, nutraceuticals as well as a wide range of chemicals. However, future attempts of engineering *S. cerevisiae*’s metabolism using synthetic biology need to move towards more integrative models that incorporate the high connectivity of metabolic pathways and regulatory processes and the interactions in genetic elements across those pathways and processes. To contribute in this direction, we have developed **M**etabolic **E**ngineering target **S**election and best **S**train **I**dentification tool (MESSI), a web server for predicting efficient chassis and regulatory components for yeast bio-based production. The server provides an integrative platform for users to analyse ready-to-use public high-throughput metabolomic data, which are transformed to metabolic pathway activities for identifying the most efficient *S. cerevisiae* strain for the production of a compound of interest. As input MESSI accepts metabolite KEGG IDs or pathway names. MESSI outputs a ranked list of *S. cerevisiae* strains based on aggregation algorithms. Furthermore, through a genome-wide association study of the metabolic pathway activities with the strains’ natural variation, MESSI prioritizes genes and small variants as potential regulatory points and promising metabolic engineering targets. Users can choose various parameters in the whole process such as (i) weight and expectation of each metabolic pathway activity in the final ranking of the strains, (ii) Weighted AddScore Fuse or Weighted Borda Fuse aggregation algorithm, (iii) type of variants to be included, (iv) variant sets in different biological levels.

**Database URL:** http://sbb.hku.hk/MESSI/

## Introduction

The suitability of *Saccharomyces cerevisiae* for the production of a range of products, such as alcohols, acids, proteins and hydrocarbons as well as pharmaceutical and nutraceutical ingredients has been demonstrated numerous times. Its attractiveness as a cell factory is mainly attributed to the fast growth on relatively cheap carbon sources, the robustness and tolerance towards harsh industrial conditions (e.g. high osmotic stress and low pH) and the well-developed genetics (1, 2). The continuous expansion of the genetic toolbox available for *S. cerevisiae* allowing manipulation of several genetic elements in a single round of transformation for strain development has placed yeast as the preferred host for bio-based production. Still, despite the several high-profile ongoing projects in both academia and industry for the use of *S. cerevisiae* to produce butanol, farnesene, stilbenes and alkaloids, to name just a few products (3), there is a clear need for the development of novel systemic approaches for the optimal—in terms of yield, productivity and final titer—functioning of the yeast metabolic network.

Metabolic engineering is exactly those integrated and multidisciplinary approaches to regulate the performance of the metabolic network for the cost-effective biological manufacturing of industrially relevant products (4–6). The field has clearly revolutionized by the explosion of information regarding metabolic pathways, not only within the genome of the host organism but essentially all organisms, the availability of ‘omic’ data and systems level modelling of function, however the integration with synthetic biology is expected to offer great power in the design of platform strains. Even though there has been a lot of debate in the definition of the fields of metabolic engineering and synthetic biology in principle the two disciplines are synergistic but use fundamentally different approaches (6). Metabolic engineering is a top-down approach for defining which pathways and in which direction should be engineered for the development of novel microbial capabilities (7). On the other hand, synthetic biology, still regarded as a young discipline, tends to be seen as a bottom-up approach for improving the design of cell factories. Propelled by the significant decrease in DNA sequencing and synthesis cost, the improved understanding on genotype-to-phenotype relationships and standardization of DNA assembly procedures, synthetic biology provides the toolbox for constructing artificial elements to achieve particular functions. Applications of synthetic biology in yeast metabolic engineering are expected to increase dramatically in the future thus development of publicly available platforms that aim to capitalize on yeast’s natural diversity for assembling biological parts with the desired properties is of utmost importance.

Following this trend we present **M**etabolic **E**ngineering target **S**election and best **S**train **I**dentification tool (MESSI), a web server for predicting efficient chassis and regulatory components for yeast bio-based production. MESSI uses publicly available metabolomic data from characterized *S. cerevisiae* strains for computing metabolic pathway activities and ranks the strains based on user-defined pathways of interest (single or multiple pathways). Furthermore utilizing the natural variation between the *S. cerevisiae* strains MESSI applies genome-wide association mapping for identifying putative genes and other genetic elements that correlate with the measured phenotype (metabolic pathway activity). MESSI is a user-friendly platform and the output generated is easy to interpret allowing the users to quickly select the most promising plug-and-play *S. cerevisiae* strain for a specific product. Candidate genes related with the pathway activity, e.g. regulatory role in controlling metabolic fluxes towards that product, are also provided.

## Materials and methods

**Figure 1.**
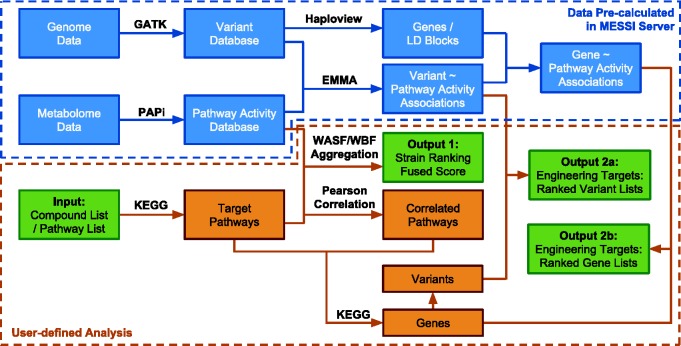
The computational pipeline of the MESSI server. Green boxes represent inputs and outputs. Data inside the blue dotted box have been pre-calculated from the exsisting database (DB01_SC_21) on the MESSI server. Steps inside the orange dotted box are user defined analysis.

MESSI implemented two major tasks. First, metabolic pathway activities were calculated based on large-scale metabolomic measurements and strain rankings based on pathway activities were further produced. Second, pathway activities and genetic variants were used to predict the potential metabolic engineering targets (variants or genes). The computational pipeline is illustrated in Figure 1. The methodology and algorithms are described in detail as follows:

### Data source and variant identification

Datasets compatible with MESSI are expected to encompass metabolomic data from large-scale genetic studies. Whole genome sequencing data are also included for predicting pathway activity associated variants and identifying metabolic engineering targets.

Since large-scale population studies of yeast with both genome and metabolome data available are still limited, we incorporated one major dataset published in 2013 (8). Based on this yeast database, 21 strains with both comparable metabolomic data and genomic data were selected and this database was named as DB01_SC_21. As pathway activities are expected to be affected by the growth conditions, including carbon source, medium, temperature and aeration conditions, all the relevant information are listed in the server. It is recommended that users apply with caution the MESSI predictions for engineering strains for which the cultivation conditions will be significantly different from the ones used in the GWAS analysis here.

To identify genetic variants (SNPs and InDels), all DNA sequencing reads were mapped to the S288C reference genome using BWA (9). Variant calling and filtering were carried out by the Genome Analysis Toolkit (GATK) (10, 11), with steps including RealignerTargetCreator, IndelRealigner, UnifiedGenotyper (parameters: -stand_call_conf 10.0 -stand_emit_conf 0 -deletions 1.0 -glm BOTH -rf BadCigar) and VariantFiltration (parameters:–filterExpression ‘ReadPosRankSum < −8.0 || FS > 10.0’). Variant effect annotation was carried out by SnpEff with the *S. cerevisiae* database version EF4.69 (12). Variants with low allele frequency (<0.05) were filtered due to possible sequencing or mapping errors, which would be detrimental to further analysis on genome-wide association and linkage disequilibrium (LD).

### Pathway activity calculation and pairwise correlations

The pathway activity profiling (PAPi) algorithm (13) was employed for calculating pathway activity based on metabolomic data. As data normalization is required by PAPi, compound concentrations were converted into ratios to the mean value of all strains. Over 100 pathway activity scores were generated for each strain. Pathways absent in Kyoto Encyclopedia of Genes and Genomes (KEGG) pathway database for *S. cerevisiae* were removed (14).

To provide users a wider range of potential metabolic engineering targets, MESSI is designed to identify variants and genes in correlated pathways, which associate significantly with the target metabolic pathway in terms of the metabolome-based pathway activities. The universal pathways, including ‘Metabolic pathways’, ‘Biosynthesis of secondary metabolites’, ‘Microbial metabolism in diverse environments’, ‘Carbon metabolism’, ‘2-Oxocarboxylic acid metabolism’, ‘Fatty acid metabolism’, ‘Biosynthesis of amino acids’, ‘Degradation of aromatic compounds’, ‘ABC transporters’ and ‘Aminoacyl-tRNA biosynthesis’, were excluded in pathway activity correlation analysis. The pairwise Pearson correlation coefficients and *P* values were calculated with R (15). *P* values were further adjusted to *Q* values using Bonferroni correction for multiple comparisons, and the significance level was set to *Q* < 0.05. Around 7% (78/1081) of the pairwise comparisons were shown significant correlation, calculated for 47 non-universal pathways with activity scores in the DB01_SC_21 database.

### Pathway activity aggregation and strain ranking

To generate a unified ranking of strains based on user-defined pathways, pathway activity aggregation is performed. Since the raw pathway activity scores (*PA*) generated from PAPi for different pathways are not comparable, they are further linearly normalized to 0–1, as normalized pathway activity scores (*PAN*), before aggregation. For strain *i* and pathway *j*, PAN*_i,j_* is calculated by the following formula:

$$PAN_{i,j}=\;\frac{PA_{i,j}\;-\;PA_{min,j}}{PA_{max,j}\;-\;PA_{min,j}}$$

For each target pathway, two parameters, pathway activity expectation (*E*) and weight (*W*), are provided from the user. Expectation (*E*) is to characterize how active the target pathway should be. Three options are available, including *Very Active*, *Medium* and *Very Weak*. Their corresponding values in the aggregation algorithm are *E* = 1, 0.5 and 0, respectively. For example, when *E* is set to 1, the strain with the highest pathway activity score (*PAN* = 1) will rank first for the selected pathway; while if it is set to 0, the strain with *PAN* = 0 will rank the first; when it is set to 0.5, the strain with *PAN* nearest to 0.5 will rank the first, and the strains with strongest or weakest pathway activity will be in the bottom. The transferred pathway activity score (*PAT*) generated for strain *i* and pathway *j* is calculated as follows:

$$PAT_{i,j}\;=\;1-\;|\;E_{j}\;-\;PAN_{i,j}\;|$$

Another important parameter, weight (*W*), is to characterize the relative importance of a particular pathway compared with other pathways in aggregation. It can be any non-negative number used as weight during aggregation. When more than one pathways are targeted, aggregation will be carried out for strain ranking. By default, universal pathways defined in the previous section, like ‘Metabolic pathway’, will receive a weight of 0 in aggregation. Users can also revise this default setting to a positive number when universal pathways are taken into consideration.

Two optional algorithms for aggregation are provided and they are the Weighted AddScore Fuse (WASF) (the default option) and Weighted Borda Fuse (WBF) (16). In the ranking process, WBF only considers pathway activity rankings (based on scores), while WASF directly uses the scores (*PAT*).

For WASF, the final fused score (*FS*) for strain *i* will be:

$$FS_{i}\;=\sum_{j}\left(PAT_{i,j}*W_{j}\right)\;$$

When WBF is used for strain *i* and pathway j, *B_i_*_,_*_j_* is the sum of strains with lower *PAT* than strain *i*. The fused score for strain *i* will be:

$$FS_{i}\;=\sum_{j}\left(B_{i,j}*W_{j}\;\right)\;$$

Finally, a strain ranking based on user-defined pathways will be generated based on *FS*.

### Genome-wide association study on pathway activities

MESSI incorporated information of compounds, pathways and genes and their relationships from KEGG database using KEGG API REST.

To identify candidate pathway activities affecting variants and genes, which could serve as potential metabolic engineering targets, an association study was performed. The variant information for different strains is retrieved and integrated from the variant calling pipelines described in the previous section. The pathway activities calculated for diversified pathways are used for different phenotypic indicators. Among the 21 strains in DB01_SC_21, GWAS was performed for 54 sets of pathway activities. The number of yeast strains used for the GWAS in the present study is comparable to previous studies reported in the literature (8, 17).

At variant (SNP or InDel) level, the efficient mixed-model association (EMMA) method was adopted to calculate genetic relatedness to pathway activities (18). As adjusted permutation test *P* values are provided by EMMA, the significance threshold is set to *P* < 0.05, for further gene-level variant statistics.

To prioritize gene level targets, gene-level association *P* values were calculated based on significant LD blocks and proxy clusters (19). LD blocks were generated by the Haploview by applying the Four Gamete Rule (20). In general, by applying this method, the most significant variant in overlapping LD blocks were considered as the true association of a gene.

Besides gene-level *P* values, other indices, such as statistics on variant quantities of diverse effects, and variant significant level accumulated score (*AS*), are also provided to users. They serve as alternative candidates for potential metabolic engineering targets. *AS_i_* is defined by the following formula:

$$AS_{i}\;=\sum_{j}\frac{-\;logP_{i,j\;}}{L_{i}}$$

Where *L_i_* stands for the length of the gene *i* region (800 bp upstream sequence is included as potential regulatory region), and *P_i,j_* stands for the *P* value for significant variant *j* in gene *i*.

## Server description

**Figure 2.**
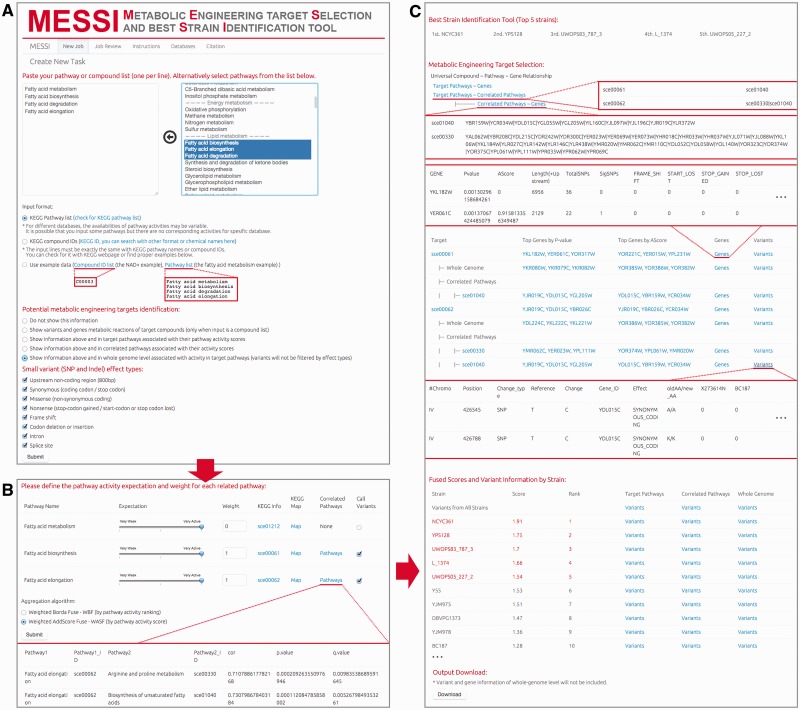
The interface of MESSI and the workflow of running a task. (**A**) Create a new task by the following steps: name a job, select a strain database, input target pathway list or compound list, and set the parameters on the potential metabolic engineering targets identification and variant calling. (**B**) Set the pathway expectations and weights and choose an aggregation algrithm. (**C**) Check for the results, including job information, best strains, metabolic engineering targets (gene-level and variant-level, respectively), fused score, ranking and variant list of each strain.

MESSI was written in R and PHP, running under the Debian 7 Linux environment. Stylesheets and functions of Shiny R package were introduced (21). There is no login requirement to use MESSI. Finished jobs are stored for two years and can be viewed using job ID. The interface and workflow of MESSI are illustrated in Figure 2. There are three main steps to run MESSI as follows:

### Input: pathway or compound list and parameters

Two input formats, KEGG pathway names and KEGG compound IDs, are acceptable in MESSI (Figure 2A). Users can select the input pathways from a candidate list on the web server. Examples for the two types of input are available for testing.

Variants and genes associated with input pathways or compounds could be summarized and demonstrated in different biological levels, serving as potential metabolic engineering targets. Optional levels include: (i) level 0, no variant and gene information will be processed; (ii) level 1, variants and genes in metabolic reactions of target compounds (only when input is a compound list); (iii) level 2, variants and genes in target pathways associated with their pathway activity score; (iv) level 3, variants and genes in correlated pathways associated with their activity scores; (v) level 4, variants and genes in whole genome level associated with activity in target pathways. When a higher level is chosen, data from lower levels are automatically processed.

Variants (SNPs and InDels) could be filtered optionally according to their mutation effects/positions, including upstream non-coding variants, synonymous coding (including synonymous amino acid coding codon or synonymous stop codon), missense coding, nonsense variants (stop-codon gained, start- or stop-codon lost situations), frame shifts, codon insertions or deletions, variants in intron and variants in splice sites.

### Second step: pathway expectation and weight adjustment

In this step, ‘Expectation’ of pathway activity, defined as how active the target pathway should be, and ‘Weight’ of pathway activity, defined as the relative importance of the pathway compared with others in aggregation, are seleted for target pathways (Figure 2B). When the input is a pathway list, all recognized pathways will be listed as target pathways. If the input is a compound list, pathways associated with target compounds in KEGG database will be listed. There are four reasons why pathways or compounds may be marked as ‘unrecognized or unused compounds or pathways’: (i) their input IDs or names could not be recognized by KEGG; (ii) the input IDs or names are recognized but are not part of the *S. cerevisiae* KEGG database; (iii) no pathway activity scores could be calculated due to data incompleteness in the metabolomic profiling of these pathways; (iv) for recognized compounds, pathway activity scores are not available for all associated pathways (similar to (iii)).

For recognized pathways, expectation (*E*) can be set as very weak, medium or very active. Weight (*W*) can be any non-negative value. Weight for universal pathways is set to 0 by default. ‘Call Variants’ should be selected if metabolic engineering targets (variants and genes) are supposed to be presented for the target pathways.

Users can also select two optional pathway activity aggregation algorithms, WBF and WASF in this step (16).

### Output: strain ranking, variants and gene-level metabolic engineering targets

In the result page, job information including job ID, input type, database and parameters are provided (Figure 2C). Based on the fused scores, the best strains (top 5) are identified and a table with scores and rankings of all strains will be listed (Figure 2C).

If the user would like to search for potential metabolic engineering targets, two types of information, variant and gene-level information, will be provided in the results. Generally, variant information may include three main parts, (i) an eight-column basic information (including chromosome, position, variant type, reference genotype, variant genotype, gene id, variant effect and amino acid alteration), (ii) a data matrix of strains (for database DB01_SC_21 it is a 21-column matrix, with 0/1 representing the variant is/is not observed in the corresponding strain, respectively) and (iii) a matrix of *P* values of associations with pathway activities. For strain-specific variant list, the data matrix of strains is not presented and only variants captured in the target strain will be listed. For pathway-specific variant list, there will be only one column of association *P* values of the specific pathway. The variant list will be sorted by *P* values, so significantly associated variants will be shown first.

Gene-level information includes gene IDs, gene-level *P* values, significance level *AS*, gene region lengths, total variant numbers, significant variant numbers and statistics for diverse effects. This information is sorted by *P* values thus genes with the highest significance levels from their LD blocks will rank first.

In the score and ranking table, strain-specific variant lists are shown in selected biological levels. In the ‘Metabolic Engineering Target Selection’ table, relationships among compounds, pathways, correlated pathways and genes are available (Figure 2C). All input and correlated sources are listed in a tree structure with hyperlinks to the corresponding KEGG information. Variant and gene lists have been generated for different sources. Top 3 associated genes of *P* value-based and *AS-*based rankings are listed, with hyperlinks to the corresponding information in Saccharomyces Genome Database (SGD) (22). Strain names are also attached with hyperlinks to the British National Collection of Yeast Cultures (23), for facilitating downstream wet-lab engineering strategies.

## Evaluation

### Experimental verification using published studies

To evaluate the reliability of MESSI in the prediction of metabolic engineering targets, we run a number of tasks and confirmed the correlations with experimental studies in the literature. All the studies presented here were performed under the same aeration condition (aerobic) and carbon source (glucose) as the strain characterization in our current pathway activity database.

Metabolic engineering to enhance fatty acid production in *S. cerevisiae* is a ‘hot’ topic for years. We created a job named ‘eg_fattyacid’, with the input pathway list including ‘Fatty acid metabolism’, ‘Fatty acid biosynthesis’, ‘Fatty acid degradation’ and ‘Fatty acid elongation’. ‘Fatty acid degradation’ was marked as unused pathway and ‘Fatty acid metabolism’ was marked as a universal pathway. For the ‘Fatty acid biosynthesis’, the pathway ‘Biosynthesis of unsaturated fatty acids’ was found as correlated pathway based on their activity scores. For ‘Fatty acid elongation’, ‘Biosynthesis of unsaturated fatty acids’ and ‘Arginine and proline metabolism’ were captured as significantly correlated. Two lists of top 3 genes (in *P* value-based or *AS*-based rankings, respectively) were generated for the two target pathways and the two correlated pathways. In a recently published study (24), eight genes were experimentally verified to be strongly correlated with fatty acid production by either gene deletion or overexpression. The genes *FAA1*, *FAA2* (24, 25), *FAS1* and *FAS2* (26) were shown to have direct correlation with fatty acid production. In *FAA1* and *FAA4* disrupted strain, approximately 80 mg/l fatty acids can be produced (25) whereas knockouts of *FAA2*, *PXA1* and *POX1*, increased the intracellular fatty acids levels by 55% (24). Similarly, the levels of lignoceric acid and cerotic acid were largely increased (40 and 50%, respectively) by the *fas1*Δ strain (26). Among these, *FAA2* and *FAA1* were successfully captured by MESSI in the top 3 in target pathway ‘Fatty acid biosynthesis’, and *POX1* was found in the top-3 in correlated pathway ‘Biosynthesis of unsaturated fatty acids’. *PXA1*, which was also experimentally shown as a regulatory point of fatty acid production, is not part of any of these pathways, however, in our analysis it was found significantly associated with the pathway activity of ‘Fatty acid biosynthesis’ and was ranked fourth in the whole-genome level statistics. *DGA1* is a similar to the *PXA1* case and was found significantly associated to the pathway ‘Fatty acid elongation’ in the whole-genome level statistics.

Another example is the NAD^+^/NADH metabolism, with the levels of NAD^+^/NADH to play important roles in yeast lifespan and have significant influence on efficient carbon source utilization (27). The job ID of this test example was ‘eg_NAD’ and the input was compound list (C00003: NAD^+^). Three target pathways, ‘Nicotinate and nicotinamide metabolism’, ‘Thiamine metabolism’ and ‘Oxidative phosphorylation’, were identified and processed. In the most relevant pathway, ‘Nicotinate and nicotinamide metabolism’, all the five genes from the two top 3 lists (with one gene shared by both rankings) have been experimentally shown to be strongly correlated with the NAD^+^ concentration [*HST1*, *HST4* (28), *PNC1* (29), *NRK1* (30) and *BNA6* (31)]. More specifically, the deletion of *HST1* resulted in up to 71% increase in NAD^+^ levels (28). On the contrary, the *Δnrk1* and *Δpnc1* double-mutant strain incapable of incorporating supplemented nicotinamide riboside (NR) into NAD^+^ in nicotinic-acid-free media (30). The *Δpnc1* mutant contributed to 69.9% decrease in the intracellular NAD^+^ concentration in stationary phase (29), while kynurenine and 3-(OH)-kynurenine in the cell, which are precursors of NAD^+^ from kynurenine pathway, were absent in the *Δbna6* mutant (31).

### *In silico* verification from Genome-scale metabolic model (GEM) simulations

We carried out *in silico* simulations to evaluate the universal gene ranking results systematically. Null mutant simulations were executed using the yeast genome-scale metabolic model *iTO977* (32) by the RAVEN Toolbox (33). The glucose uptake rate was set to 10 mmol/g/h and the simulations were optimized for maximum growth. For the original model and each single gene deletion simulations, all reaction fluxes were calculated and then summarized by individual non-universal pathways. Reactions without any perturbation in all simulations, and pathways without valid activity information or with less than 6 simulated reactions were removed in the statistical analysis. 40 pathways were processed, with 21.5 genes, 28.8 reactions and 10.7 reserved reactions on average. We assumed the fluxes of a reaction among all 977 single gene deletion simulations have a normal distribution (with the population mean the flux of the original model) thus we converted all fluxes into *Z*-scores and calculated their *P* values. The proportions of significantly altered fluxes (*P* < 0.05) were compared between the top 3 genes from the *P* value-based ranking, and the rest of the genes for each pathway, respectively. It was observed that the proportions of significantly altered fluxes of the top ranked genes are significantly higher than the rest ones in single gene deletion simulations (3.49% versus 1.87, *T*-test *P* = 0.022), illustrating the potential regulatory role of the MESSI output.

## Conclusions and future directions

The ability to perform deep sequencing of industrially relevant microbial species at increasingly affordable costs can help to revolutionize microbial cell factory engineering in a similar way that revolutionized fields like human genetics and epigenetic studies. With this in mind, we developed MESSI, a *S. cerevisiae* web server, where we incorporated bioinformatics methods—that are being employed in NGS-based human genetics—for prioritizing genetic changes that need to be experimentally tested. The ultimate goal of MESSI is to provide a more solid and comprehensive basis for selecting the most promising host for desired phenotypes and discover which mutations would be expected to contribute most to that phenotype for metabolic engineering efforts. We believe that MESSI offers new opportunities for establishing links between genotype and phenotype in *S. cerevisiae* strains and can be efficiently used for searching genome-wide spaces for small variants and genes conferring phenotypic characteristics of interest.

The first version of MESSI contains 21 *S. cerevisiae* strains for best strain selection and genotype-to-phenotype mapping. Even though the number of yeast strains is comparable with other studies in the literature (8, 17) we intend to significantly expand the *S. cerevisiae* strain database to achieve higher confidence in the GWAS mapping and improve the prediction of regulatory points in the different metabolic pathways. Towards that objective we have initiated a collaborative effort to sequence the genomes and perform metabolomic profiling of >35 *S. cerevisiae* strains, including several industrial strains. To deal with the limitation of KEGG database and the ready-made definition of its pathways, a further direction of MESSI may be a comprehensive expansion of pathway databases (for instance, Reactome (34), SGD (22), *etc.*). Furthermore, based on the better-defined pathways and the *in-house* strain set, we will work on an algorithm development to compute the optimized pathway expectation (*E*) and weights (*W*) automatically from the pathway topology, and then evaluate the method with downstream engineering in our strain set. Last but not least, we will continuously add more strain databases from different cultivation conditions, for instance, diverse carbon sources (ethanol is next in the pipeline) and cultivation conditions (batch cultivation, anaerobic, *etc.*), to improve the practical value of MESSI in industrial bioengineering.
